# Changes in functional status during and after pregnancy in people with cerebral palsy: An international observational study

**DOI:** 10.1177/1753495X241297560

**Published:** 2024-11-13

**Authors:** Georgia Condran, Hayley Lipworth, Anne Berndl

**Affiliations:** 1 Temerty Faculty of Medicine, 7938 University of Toronto, Toronto, ON, Canada; 2 7938 University of Toronto, Toronto, ON, Canada; 3 71545 Department of Obstetrics and Gynaecology, Division of Maternal-Fetal Medicine, Sunnybrook Health Sciences Centre, University of Toronto, Toronto, Canada

**Keywords:** Pregnancy, cerebral palsy, disabled persons, postpartum period

## Abstract

**Background:**

There is limited information surrounding physical changes people with cerebral palsy (CP) experience during pregnancy.

**Methodology:**

This is a subgroup analysis of an international online questionnaire, developed with input from individuals with CP. Data collection included demographics, baseline functional status, and functional status changes during pregnancy. Descriptive analysis was used.

**Results:**

158 participants from 15 countries participated of which 30 had a total of 49 pregnancies resulting in birth. Worsened strength, spasticity, bladder function and fatigue occurred in 56.5% (13/23), 47.8% (11/23), 56.5% (13/23) and 87.0% (20/23) of participants. 9/23 (40.9%) required new mobility devices. Worsening of fatigue, strength, spasticity and need for a new mobility device was reported by all groups regardless of functional status.

**Conclusions:**

People with CP may experience significant functional changes during pregnancy, even those who mobilized independently prior to pregnancy. These findings may inform obstetricians, nurses, neurologists and physiatrists, and contribute to preconception counselling.

## Background

Cerebral palsy (CP) is a common disability with high rates of comorbidities and lifelong complications.^1^ A recent systematic review by McIntyre et al. found that the prevalence of CP is as high as 1.6 per 1000 live births in high income countries, and 3.4 per 1000 live births in low- and middle-income regions.^2^ The prevalence in high income countries appears to be decreasing. CP is a motor function disorder that occurs due to insult to the developing brain, usually due to perinatal circumstances including birth asphyxia, fetal growth restriction, trauma, infection or preterm delivery.^3^ Its clinical manifestations depend on area of brain involvement and can be either spastic, dyskinetic, ataxic or mixed types.^4^ This variety of presentations may impact strength, spasticity, continence, speech, mobility, seizures and/or cognition, among other changes in functional status. Individuals with CP are at increased risk of a variety of health conditions including pain disorders, asthma, obesity and epilepsy.^5^

The intersection of CP and pregnancy remains a limited area of research, however understanding the impact of pregnancy on the CP-related health of an individual is important for preconception, prenatal, antepartum and postpartum counselling. Due to advances in healthcare, more individuals with CP are living full, healthy lives and are reaching reproductive age.^6,7^ More individuals with disabilities may contemplate pregnancy, making accessible reproductive healthcare an increasingly important area.^8,9^ Research has shown that 1 in 9 individuals with CP have biological children.^10^ A previous study found that pregnancy in people with CP was associated with an almost 3-fold increased risk for preterm birth, 2-fold increased risk of lower (uterine) segment caesarean section, and low 5-min Apgar score.^1^ It is felt that the unique pregnancy experiences of people with disabilities, including a high degree of mobility changes, such as pain and spasticity,^11^ as well as other key quality of life parameters such as bladder and bowel function may impact care priorities in this population.^8,12^ The time to recover after birth is currently unknown.

These pregnancy changes may have vast psychological and social implications for the postpartum period including impact on breastfeeding, newborn bonding and postpartum depression.^9,13^ It is crucial to investigate how CP impacts an individual and recognize that CP comorbidities during pregnancy and recovery may affect people differently. The needs of individuals during pregnancy and following delivery may vary depending on the changes in functional status that they experienced and their risk of increased disability in the postpartum period.

This overall lack of research may impact the care received among pregnant people with CP. The objective of the current study was to utilize data obtained through a large international survey investigating CP and reproductive health to examine changes in functional status during pregnancy and time to recovery in this population. The aim of this study was to provide clinical information for people with CP who are contemplating pregnancy or currently pregnant, as well as obstetricians, nurses, physiatrists and neurologists.

## Methods

This research is a sub-analysis of an international observational questionnaire examining perinatal outcomes of pregnant people with CP. This study obtained research ethics approval through Sunnybrook Health Sciences Centre (REB number: 3048-2018). Individuals with CP were included in the survey development to ensure a person-centred approach and the online questionnaire was distributed electronically to multiple international CP organizations. Snowball and peer recruitment were utilized. To be included in this sub-study of the larger questionnaire, participants needed to speak English, be age 18 and over, be assigned female at birth, have a self-reported diagnosis of CP, and experience of a pregnancy that resulted in a live or stillbirth. The outcomes measured in this study included participant demographics (e.g. age, ethnicity, CP diagnosis, gross motor function classification system (GMFCS) level and the manual ability classification system (MACS) level), obstetric outcomes, changes in functional status during pregnancy and time to full recovery. Descriptive analysis of each pregnancy was used. Thirty people had a total of 49 pregnancies that resulted in still or livebirth. These 30 individuals responded to optional questions about experiences during pregnancy in the online questionnaire.

## Results

A total of 158 participants from 15 countries with CP were included in this sub-analysis of the international observational questionnaire. The participation rate was 67.8% (158/233). In this study, 30 people had a total of 49 pregnancies that resulted in still or livebirth. Not every participant who had a pregnancy responded to every question, therefore, every pregnancy did not respond to every question. Only answered questions were used in the analysis. Therefore, the denominators for the measured outcomes are different.

The demographic characteristics of participants in this study are summarized in Table 1. Participants in this study were from high-income countries, and more than 80% were of white ethnicity. Most CP diagnoses were diplegia (14/30), hemiplegia (8/30) and spastic palsy (19/30); more than one description could be selected.

**Table 1.** Demographic data of people with cerebral palsy who had either a live or stillbirth.

Variable	Participants (*n* = 30) No. (%)
Age at study completion (years)	41.1 ± 11.2
Age at first pregnancy (years)	27.1 ± 6.4
Country	
Canada	9 (30.0)
United States	18 (60.0)
United Kingdom	2 (6.7)
Norway	1 (3.3)
Ethnicity	
White	25 (83.3)
Black	1 (3.3)
Southeast Asian	1 (3.3)
/West Asian	1 (3.3)
Other	2 (6.7)
Cerebral palsy diagnosis	
Hemiplegia	8 (26.7)
Diplegia	14 (46.7)
Tetraplegia/quadriplegia	2 (6.7)
Monoplegia	0 (0)
Triplegia	1 (3.3)
Spastic CP	19 (63.3)
Ataxia	7 (23.3)
Dyskinesia	6 (20.0)
Mixed	3 (10.0)
Gross Motor Function Classification System (GMFCS)	
GMFCS Level I	5 (16.7)
GMFCS Level II	13 (43.3)
GMFCS Level III	3 (10.0)
GMFCS Level IV	2 (6.7)
GMFCS Level V	1 (3.3)
Other	6 (20.0)
Manual Ability Classification System (MACS)	
MACS Level I	9 (30.0)
MACS Level II	20 (66.7)
MACS Level III	1 (3.3)
MACS Level IV	0 (0)
MACS Level V	0 (0)
Use of mobility device	
No device	6 (20.0)
Cane, brace, or walker	11 (36.7)
Wheelchair (manual or power) at least some of the time	13 (43.3)
Expressive communication skills	
Speak with no difficulty	18 (60.0)
Speak with minor limitations	8 (26.7)
Speak with some difficulty	3 (10.0)
Speak with very significant difficulty	1 (3.3)
Pregnancy outcome	Births (*n* = 49)
Live birth	48 (96.0)
Still birth	1 (2.0)
Mode of delivery	Births (*n* = 49)
Vaginal birth	31 (63.3)
Caesarean delivery	18 (36.7)

Of the 49 births included in this analysis, 1 (2.0%) resulted in stillbirth and 48 (96.0%) in live births, with the majority delivering vaginally (63.3% of live births).

When examining gross motor abilities based on the GMFCS, 16.7% (5/30) of participants reported being able to walk without limitations, 43.3% (13/30) of participants reported being able to walk with some limitations without an assistive device, and only one individual reported that they usually use a wheelchair and have difficulty keeping their head and trunk upright. Further analysis with the MACS revealed that most individuals reported being able to handle most objects, but with reduced quality and speed of achievement (66.7%). The majority of individuals required a mobility device such as a cane, brace or walker (36.7%) or a wheelchair (43.3%).

**Figure 1.**
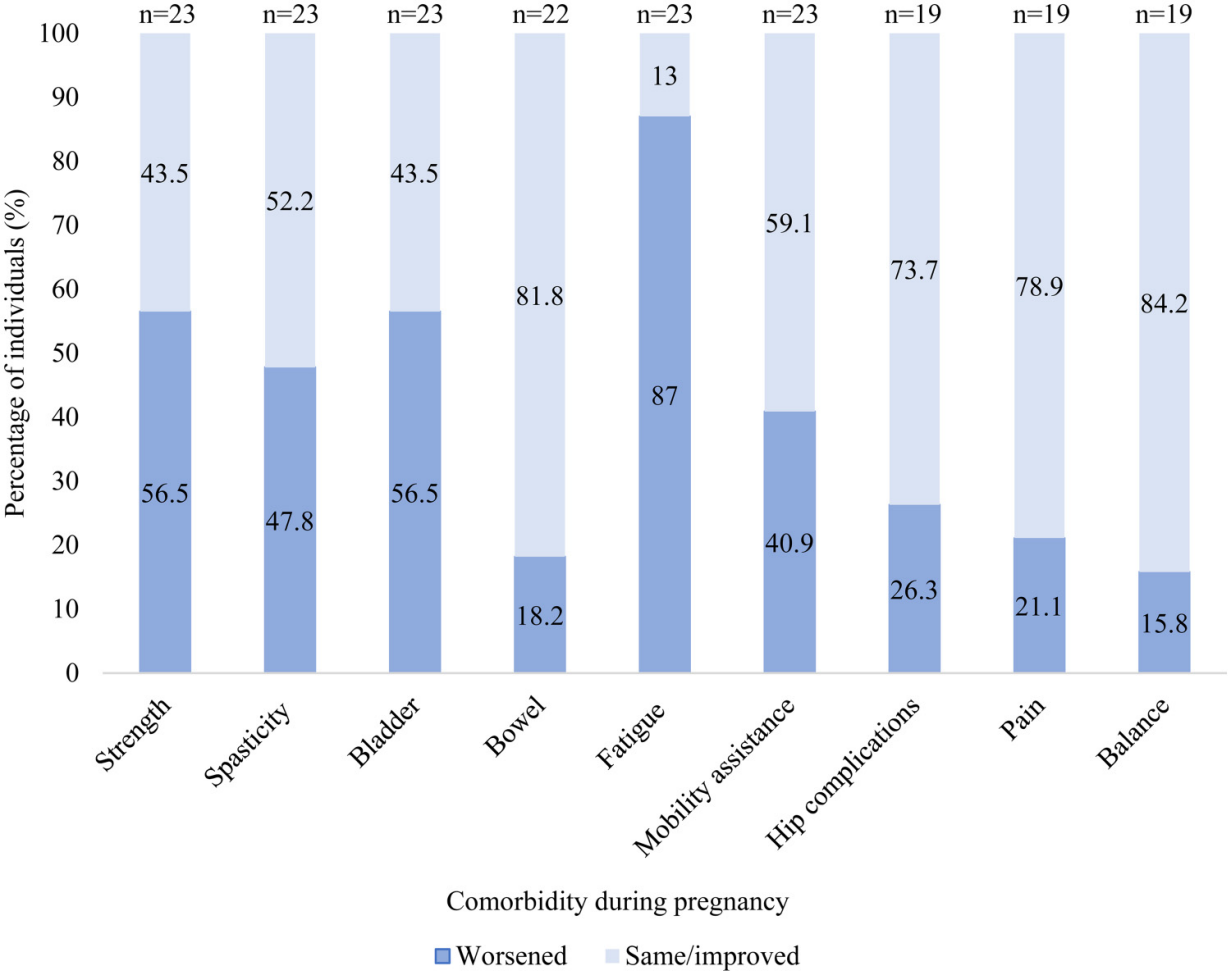
Changes in functional status experienced in pregnancy by people with CP.

Changes in functional status experienced by individuals with CP during pregnancy are summarized in Figure 1. 22/23 reported at least one worsening symptom during pregnancy with the most common being worsening fatigue. Worsening symptoms were reported by people with all GMFCS categories.

Time to full recovery is described in Table 2. Full recovery was defined as a return to pre-conception functional status. By one year, 70% (16/23) of people felt they had recovered.

**Table 2.** Time to recovery following delivery in people assigned female at birth with cerebral palsy.

Variable	Participants (*n* = 23) % (No.)
Recovery within 2 weeks	8.7 (2/23)
Recovery within 6 weeks	13.0 (3/23)
Recovery within 6 months	13.0 (3/23)
Recovery within 1 year	34.8 (8/23)
Had not recovered by 1-year post-partum	30.4 (7/23)

## Discussion

In the past, stigma associated with disability and sexuality limited childbearing in this population.^12,14^ However, greater recognition of reproductive health in people with disabilities and the knowledge that fertility is not impacted by CP,^15^ has led to a greater appreciation that these individuals can have successful pregnancies.^16,17^ In reality, many pregnant people with CP continue to find it challenging to find adequate antenatal care that meets their unique needs, including adequate access to care, physically accessible care environments and appropriate approaches to care.^12,18,19^ This issue may be perpetuated by a continued lack of rigorous studies on changes in functional status during pregnancy and time to full recovery in individuals with CP, which may shape conversations with physicians about pre-pregnancy counselling, antepartum and postpartum management, and care received.

This study aimed to examine pregnancy outcomes in individuals with CP to understand the physical changes during pregnancy and in the postpartum period in this population. Analysis revealed that pregnant people with CP face many unique challenges including worsening of strength, spasticity, pain and fatigue, as well as mobility changes with the vast majority (22/23) experiencing at least one worsening symptom. Interestingly, worsening fatigue, strength, spasticity and a need to change or get a new mobility device occurred in all GMFCS categories, indicating that all individuals with CP, regardless of prior need for mobility device, may experience these changes in functional status, although numbers are too small to calculate differences in this outcome between groups. In addition, results highlighted heightened risk of bowel and bladder concerns. While there is limited research on changes in functional status during pregnancy and CP specifically, these findings are consistent with currently available data which suggests that rates of pregnancy related comorbidities may be higher in individuals with physical disabilities broadly, primarily decreased mobility and bladder concerns.^20^ The increased rate of bladder concerns during pregnancy in those with pre-existing bladder dysfunction is potentially due to the pressure of the growing fetus.^21^ Iezzoni et al. (2014) report that people with mobility-associated disability experienced increased rates of impairment-related complications during pregnancy including bladder problems, wheelchair fit problems and reduced mobility, increased spasticity and bowel management difficulties.^11^ These authors concluded that changes reported by those with mobility disabilities likely differed substantially from experiences of pregnant people without disabilities. Another study examining approaches to care for pregnant people with disabilities suggests an increased prevalence of urinary tract infections, urinary incontinence, spasticity, constipation and unrecognized onset of labour.^9^ Smeltzer (2007) also reported an increase in severe fatigue and risk of falls in pregnant individuals with physical disabilities. These findings highlight the considerations to be made when providing antenatal care for this population, suggesting the potential benefits of multidisciplinary care teams, including obstetricians, nurses, neurologists, physiatrists, family physicians, nurses, occupational and physical rehabilitation specialists.

The time to recovery of preconception functional status following pregnancy in people with physical disabilities, including CP, appears to be higher than the general population.^9^ Smeltzer (2007) found that there may be prolonged recovery from birth experience and individuals with physical disabilities may require longer hospital stays to recover from vaginal or caesarean delivery. This may be compounded by a lack of specialized rehabilitation and support services designed to meet the unique needs of this population.^9,21^ A recent study from the United States highlighted the unmet needs of individuals with heterogeneous physical disabilities during pregnancy and childbirth. The authors reported that there is a lack in clinician knowledge, physically inaccessible care facilities and a need for research related to disability in pregnancy.^22^

The strengths of this study lie in the inclusion of detailed demographics of the functional abilities of participants using the GMFCS and MACS, as well information related to the use of mobility equipment. As well, it details some of the specific changes in functional status that may be anticipated during pregnancy, which may require different approaches to management.

This study is subject to limitations inherent in survey research. Standardization and measurement issues may arise from variation in how questions are interpreted and answered by participants. Variation in participant engagement and honesty may further complicate the reliability of findings. Response and sampling biases may influence results, especially given the small sample size of the study, which may not be representative of the full spectrum of CP. For example, people choosing to participate in this study may be more impacted by their CP diagnosis than the whole population, as an individual less impacted by CP may be less likely to be connected to CP-related websites, where this study was primarily advertised. The analysis may also not include individuals most impacted by their CP who may not be able to complete an online questionnaire. Further, this study was limited to individuals in high-income regions who may have better access to medical and allied health professional care. It is possible that people with CP who attempt pregnancy have less severe impairment than the average person with CP. The majority of participants in this study were in GMFCS levels I and II (60.0%). These rates are similar to studies of motor severity levels in the general CP community in Australia and Sweden (59.8% and 61.0%, respectively).^23,24^ A study on hand function in people with CP found that 42.2% of participants were in MACS level I.^25^ This is similar to the sample in the present study, 30.0% of whom were classified as MACS level I. Therefore, the current study appears to be reflective of the overall CP population from a functional perspective. Although, the small sample size prevents meaningful comparison between GMFCS and MACS groups. Another limitation is that the survey had non-mandatory questions and analysis was completed on pregnancies with response.

## Conclusion

This study has demonstrated that individuals with CP, even those with minimal mobility concerns, appear to be at risk of CP-specific changes in functional status during pregnancy. This is an important finding for pregnant people with CP to both prepare for and manage. For example, all people with CP regardless of pre-existing functional status may benefit from counselling surrounding need for a new or modification of mobility device and a discussion surrounding pain management and fall prevention. These changes in functional status may persist after childbirth. This information may inform obstetricians, nurses, neurologists, physiatrists, physiotherapists and others who provide care for people with CP, and may inform preconception counselling as well as management during pregnancy and beyond.
